# High Concentrations of Immunoglobulin G Against Cow Milk Proteins and Frequency of Cow Milk Consumption Are Associated With the Development of Islet Autoimmunity and Type 1 Diabetes—The Trial to Reduce Insulin-dependent Diabetes Mellitus (IDDM) in the Genetically at Risk (TRIGR) Study^☆^^☆☆^

**DOI:** 10.1016/j.tjnut.2024.06.005

**Published:** 2024-06-19

**Authors:** Sari Niinistö, David Cuthbertson, Maija E Miettinen, Leena Hakola, Anita Nucci, Tuuli E Korhonen, Heikki Hyöty, Jeffrey P Krischer, Outi Vaarala, Mikael Knip, Erkki Savilahti, Suvi M Virtanen, TRIGR Investigators

**Affiliations:** 1 Department of Public Health and Welfare, Finnish Institute for Health and Welfare, Helsinki, Finland; 2 Health Informatics Institute, Morsani College of Medicine, University of South Florida, Tampa, FL, United States; 3 Unit of Health Sciences, Faculty of Social Sciences, Tampere University, Tampere, Finland; 4 Tampere University Hospital, Wellbeing Services County of Pirkanmaa, Tampere, Finland; 5 Department of Nutrition, Georgia State University, Atlanta, GA, United States; 6 Faculty of Medicine and Health Technology, Tampere University, Tampere, Finland; 7 Fimlab Laboratories, Pirkanmaa Hospital District, Tampere, Finland; 8 Research Program for Clinical and Molecular Metabolism, Faculty of Medicine, University of Helsinki, Helsinki, Finland; 9 Pediatric Research Center, New Children’s Hospital, Helsinki University Hospital, Helsinki, Finland; 10 Department of Pediatrics, Tampere University Hospital, Tampere, Finland; 11 Center for Child Health Research, Tampere University and Tampere University Hospital, Tampere, Finland

**Keywords:** cow milk antibodies, islet autoimmunity, infants, children, type 1 diabetes, extensively hydrolyzed infant formula, cow milk–based infant formula, cow milk

## Abstract

**Background:**

The Trial to Reduce IDDM in the Genetically at Risk (TRIGR) (NCT00179777) found no difference type 1 diabetes risk between hydrolyzed and regular infant formula. However, cow milk consumption during childhood is consistently linked to type 1 diabetes risk in prospective cohort studies.

**Objectives:**

Our primary aim was to study whether humoral immune responses to cow milk and cow milk consumption are associated with type 1 diabetes in TRIGR children.

**Methods:**

TRIGR comprised 2159 children with genetic susceptibility to type 1 diabetes born between 2002 and 2007 in 15 countries. Children were randomly assigned into groups receiving extensively hydrolyzed casein or a regular cow milk formula and followed up until age 10 y. Type 1 diabetes-related autoantibodies and antibodies to cow milk proteins were analyzed. Infant formula intake was measured by structured dietary interviews and milk consumption with a food frequency questionnaire. Associations of milk antibodies and milk consumption with risk to develop type 1 diabetes were analyzed using Cox survival model.

**Results:**

Cow milk antibody concentrations both in cord blood [hazards ratio (HR) for islet autoimmunity: 1.30; 95% CI: 1.05, 1.61; HR for type 1 diabetes: 1.32; 95% CI: 1.02, 1.71] and longitudinally from birth to 3 years (HR for islet autoimmunity: 1.39; 95% CI: 1.07, 1.81; HR for type 1 diabetes: 1.43; 95% CI: 1.04, 1.96) were associated with increased risk of developing type 1 diabetes. The amount of regular infant formula was associated with reduced islet autoimmunity risk in the regular infant formula group (HR: 0.92; 95% CI: 0.85, 0.99). Furthermore, frequent liquid milk consumption after infancy was associated with increased risk of islet autoimmunity or type 1 diabetes.

**Conclusions:**

Elevated cow milk antibody concentrations and high consumption of liquid milk after infancy are related to type 1 diabetes development in children with an increased genetic susceptibility to type 1 diabetes. Enhanced antibody concentrations to cow milk may provide a biomarker of immune system prone to develop islet autoimmunity.

This trial was registered at clinicaltrials.gov as NCT00179777.

## Introduction

Type 1 diabetes is an autoimmune disease in which the immune system erroneously attacks and destroys the insulin-producing pancreatic β cells. Impaired tolerance to dietary proteins may be a factor in the etiology of type 1 diabetes. Cow milk has been suggested to be 1 trigger of the disease process leading to type 1 diabetes. Enhanced humoral immune responses to cow milk proteins were associated with increased risk of type 1 diabetes in the small TRIGR pilot study in children with an increased genetic susceptibility to type 1 diabetes (*n* = 94) [1] and several case-control studies [[2], [3], [4], [5], [6], [7], [8], [9], [10], [11]]. Cow milk–based infant formula feeding or intake of other dairy products correlated directly with antibodies to cow milk protein [3,5,9,11], whereas breastfeeding correlated inversely [3,11].

It has been suggested that children who develop type 1 diabetes have enhanced immunologic reactivity or gut permeability to cow milk proteins or altered cow milk consumption [12]. Prospective findings regarding whether age at exposure to cow milk products is associated with risk of type 1 diabetes are inconsistent [13]. However, the amount of cow milk consumed during childhood has been consistently shown as a risk factor for islet autoimmunity and type 1 diabetes in children with an increased genetic susceptibility to type 1 diabetes [[13], [14], [15], [16], [17], [18], [19]]. It is unclear which factor in cow milk, if any, affects risk of development of type 1 diabetes. In the TRIGR pilot study, extensively hydrolyzed infant formula compared with a regular cow milk–based formula protected from islet autoimmunity [20], whereas in the completed TRIGR trial, no effect was seen [21]. In the Finnish Dietary Intervention Trial for the Prevention of Type 1 Diabetes pilot trial among children with genetic susceptibility to type 1 diabetes insulin-free formula protected from islet autoimmunity compared with regular cow milk–based formula, suggesting that immunization against cow milk insulin may be an important pathogenic factor [22].

Our primary aim was to investigate prospectively whether humoral immune responses to cow milk and cow milk intake are associated with risk of development of type 1 diabetes in a large, international infant feeding trial series in children with an elevated genetic susceptibility to type 1 diabetes. Our hypothesis was that increased immune responses to cow milk and high cow milk consumption are associated with higher risk of islet autoimmunity and type 1 diabetes. In addition, we wanted to study associations of infant milk feeding and later consumption of dairy foods with concentrations of milk antibodies to understand their role as markers of milk consumption.

## Methods

### Study design and participants

TRIGR is an international double-blind randomized clinical trial (NCT00179777) of 2159 infants with human leukocyte antigen (HLA)-conferred genetic disease susceptibility and ≥1 first-degree relative with type 1 diabetes, recruited between 2002 and 2007 in 15 countries followed up until the youngest child turned 10 y of age [21]. In the current data set of 2069 children, 172 (8.3%) had developed type 1 diabetes. The inclusion criteria of the TRIGR study encompassed both certain HLA-conferred genotypes known to increase risk of type 1 diabetes and a first-degree relative affected by type 1 diabetes [21]. TRIGR tested whether weaning to an extensively hydrolyzed casein infant formula compared with a regular cow milk–based one (control) during the first 6–8 mo of life protected from type 1 diabetes. Randomization stratified by geographic area was implemented after 35 wk of gestation or immediately after birth within 7 d of delivery. Ethical approval was obtained at each study center. Exclusion criteria included multiple gestation, an older sibling already participating in TRIGR, recognizable severe illness, gestational age <35 wk, age of the infant >7 d at randomization, and no HLA sample drawn before the age of 8 d. Recruitment and retention strategies have been described in detail previously [23]. The flow chart illustrating the screening, randomization, and follow-up of infants involved in the TRIGR study, along with the reasons for nonparticipation at each stage, is presented in Supplemental Figure 1. Cow milk antibodies could be measured from 2069 children (96% of the whole TRIGR cohort). The parents of 1506 children filled in questionnaire of cow milk consumption after infancy (70% of the whole TRIGR cohort).

### Dietary intervention and collection of dietary data

Breastfeeding was encouraged. Infants were randomly assigned in a double-blind fashion to receive either an extensively hydrolyzed casein–based infant formula or a control infant formula on weaning from breast milk or when supplemental feeding was needed. Both study formulas were nutritionally complete infant formulas in powder form manufactured by 1 company and provided to families free of charge (Mead Johnson Nutritionals). The control formula was a mixture of commercial regular cow milk–based formula powder plus casein hydrolysate powder in a 4:1 ratio to mask the flavor and smell differences between the formulas. Extensively hydrolyzed casein formula Nutramigen or banked breast milk was used if feeding other than breast milk was needed before randomization or in delivery hospitals if study formula was unavailable to avoid exposure to intact cow milk proteins. The intervention lasted until infants were 6 mo old or if they had not received study formula for ≥60 d at that time until the age of 7–8 mo. Families were counseled to avoid all other infant formulas and food products containing cow milk or beef. Nutramigen was given to infants with suspected or proven cow milk allergy. The use of any other infant formula (e.g., soy-based ones) was discouraged, to maximize exposure to study formula. Parents were provided lists of foods with brand names that did not contain cow milk protein and could be used during the intervention as well as lists of nonrecommended foods containing dairy protein. Noncompliance was defined as any reported exposure to nonapproved formula (anything but assigned study formula or Nutramigen) or nonrecommended foods (any foods containing milk or beef proteins). We used infant feeding information that was acquired from the family through standardized dietary interviews at the age of 3 and 6 mo. Amount of study formula given and returned was registered and cumulative amount consumed by the infant during the first 6 mo was calculated. The children’s later consumption of dairy products was assessed with a food frequency questionnaire biannually from the age of 18 mo to 10 y of age. Liquid milk variable included consumption of drank cow milk as such but not milk from other drinks or foods. Sour milk products consisted the use of e.g., yogurt, buttermilk, acidophilus milk, and Lactaid milk. Cheeses variable included the use of e.g., cheese or cheese spread, cream cheese, cottage, or ricotta cheese.

### Assessment of HLA genotype, type 1 diabetes-related autoantibodies, and cow milk antibodies

HLA genotyping for the selected *DQB1* and *DQA1* alleles was performed using sequence-specific oligonucleotide hybridization and genotypes categorized as high, moderate, and mild risk of type 1 diabetes.

Type 1 diabetes-related autoantibodies (autoantibodies to glutamic acid decarboxylase, insulinoma-associated antigen 2 antibody, insulin, and, and zinc transporter 8) were measured by specific radiobinding assays and islet cell autoantibodies by conventional immunofluorescence from blood samples collected at 3–12 mo of intervals as described [20]. The definition of outcomes were following: repeated positivity for ≥2 autoantibodies of the following—islet cell autoantibodies, insulin autoantibody, insulinoma-associated antigen 2 antibody, glutamic acid decarboxylase, and zinc transporter 8 antibody (persistent multiple islet autoimmunity)—and clinical type 1 diabetes.

IgA and IgG antibodies to cow milk formula and α-casein, were measured from serum samples obtained from cord blood and at the age of 3, 6, 9, 12, and 18 mo and 2 and 3 y by ELISA, as described previously [4,24]. In brief, microtiter plates (Linbro; Flow Laboratories) were coated with either diluted or defatted (1:500) adapted liquid cow milk formula (Tutteli; Valio) at a concentration of 1 μg/mL in carbonate buffer, pH 9.6, overnight. Diluted (1:40 for cow milk) sera was applied in triplicate to the antigen-coated plates and in duplicate to wells of the same microtiter plates coated with blocking solution (1% sheep serum); the plates were incubated overnight at room temperature. After washing, 75 μL alkaline phosphatase–conjugated mono-specific swine antihuman IgG and IgA antisera (diluted 1:200; Orion Diagnostica) were added, and the plates were incubated for 60 min at 37 °C. After washing, 75 μL of p-nitrophenylphosphate substrate (2 mg/mL in diethanolamine buffer, pH 10.0; IT Baker Chemical) was added. The reaction was stopped after 30 min with 75 μL of 1 M NaOH. The end product was measured at 405 nm in a semiautomatic photometer (Titertek Multiscan; Elflab). The mean value of the 2 absorbencies for the wells coated with blocking solution was subtracted from the mean value for the 3 absorbencies in the antigen-coated wells. Antibody values were expressed as percentages of the standard with a very high titer of cow milk antibodies [4].

### Statistical analyses

Associations between cow milk antibody concentrations and consumption of infant formula as well as later intake frequency of dairy products with risk of multiple islet autoimmunity and type 1 diabetes were analyzed using Cox time-dependent survival model. We adjusted the Cox models for sex, HLA, region, maternal type 1 diabetes status (categorized as in Table 1), and weight *z*-score. Weight *z*-score was obtained from Centers for Disease Control and Prevention standardized growth charts. Serum cow milk antibody concentrations were transformed using LOG10(*x* + 1) transformation. Thus, 1-unit change in hazards ratio (HR) represents 10-fold increase in milk antibody value. Observations with missing values (either outcome or explanatory parameters) were excluded from the Cox regression analyses. For the Cox modeling, the assumption of proportional hazards for all time-dependent variables was reviewed using graphical means and tested for linearity. The assumption of proportional hazards failed to be rejected for all models. For children aged 3 and 6 mo, Spearman correlation coefficients between consumption of infant formula and milk antibodies at respective age were calculated separately by breastfeeding and by treatment group (casein hydrolysate and control formula). Spearman correlations between intake frequency of later milk product and milk antibodies at respective ages of 18 mo and 2 and 3 y were analyzed for both treatment groups combined. Differences between milk antibody concentrations by treatments and control formula groups were tested by Wilcoxon Rank Sum test. No adjustments were made for multiple testing. All analyses were performed using SAS v9.4. Statistical significance was determined when the *P* value was <0.05.

**TABLE 1:** Characteristics of TRIGR study participants.

	Cow milk antibody measurement (*N* = 2069), *n* (%)	Milk consumption data after infancy (*N* = 1506), *n* (%)
Sex		
Boys	1092 (52.8)	810 (53.8)
Girls	977 (47.2)	696 (46.2)
HLA risk^1^		
High risk	496 (24.0)	366 (24.3)
Moderate risk	911 (44.0)	664 (44.1)
Mild risk	662 (32.0)	476 (31.6)
Family member with type 1 diabetes		
Mother alone or with father or sibling	1026 (49.6)	735 (48.8)
Father or/and full sibling	1043 (50.4)	771 (51.2)
Treatment group in TRIGR study		
Casein hydrolysate	1038 (50.2)	755 (50.1)
Control formula	1031 (49.8)	751 (49.9)
Region		
Finland	412 (19.9)	391 (26.0)
Canada	502 (24.3)	345 (22.9)
United States	386 (18.7)	331 (22.0)
Other	769 (37.2)	439 (29.2)
Any breastfeeding		
<3 mo	600 (29.0)	421 (28.0)
3–5.9 mo	267 (12.9)	178 (11.8)
≥6 mo	1202 (58.1)	907 (60.2)

1 High risk: HLA-DQB1∗0302/DQB1∗02; moderate risk: HLA-DQB1∗0302/x (x not DQB1∗02, DQB1∗0301, or DQB1∗0602); mild risk: HLA-DQA1∗05-DQB1∗02/y (y not DQA1∗0201-DQB1∗02, DQB1∗0301, DQB1∗0602, or DQB1∗0603) and HLA-DQA1∗03-DQB1∗02/y (y not DQA1∗0201-DQB1∗02, DQB1∗0301, DQB1∗0602, or DQB1∗0603).

## Results

### Cow milk antibodies and risk of multiple islet autoimmunity and type 1 diabetes

**FIGURE 1:**
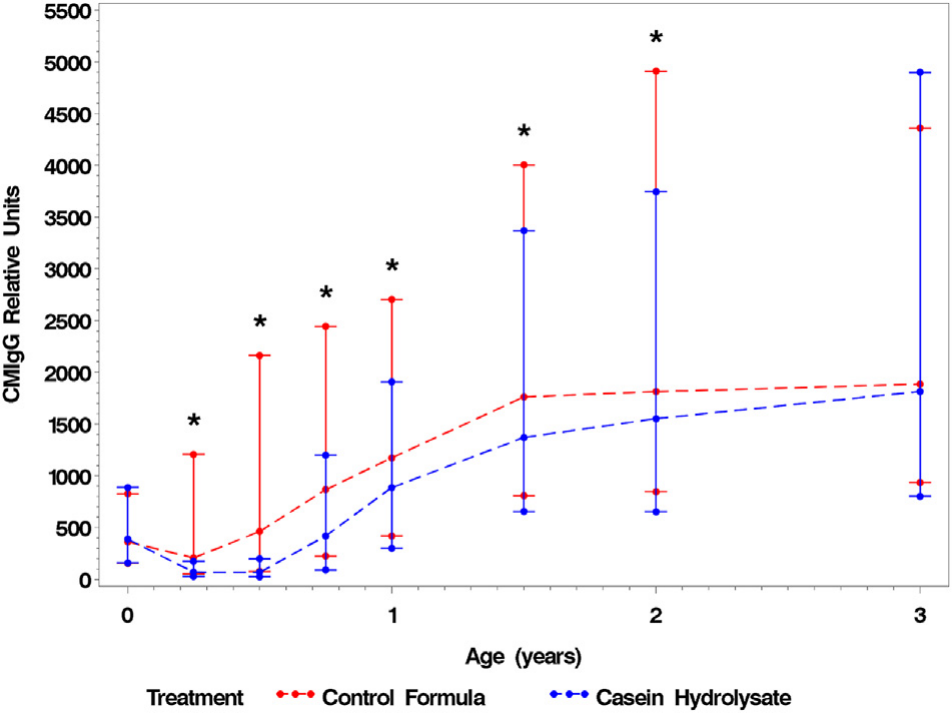
Median and IQR of cow milk IgG (CMIgG) concentrations by age and treatment group. Differences between milk antibody concentrations by casein hydrolysate and cow milk–based control formula groups was tested using Wilcoxon rank sum test (∗*P* < 0.05).

Characteristics of children are presented in Table 1 for those children from whom milk antibodies were measured and for those from whom information on dairy product intake frequency between 1.5 and 10 y was available. Milk antibodies were analyzed from all those children from the whole cohort who had blood sample available for milk antibody analysis. Cow milk IgG concentrations were higher in control formula group than those in the casein hydrolysate group from 3 mo until 2 y of age (Figure 1). Increased concentrations of cow milk IgG from birth to 3 y of age was associated with increased risk of developing multiple islet autoimmunity (HR: 1.39; 95% CI: 1.07, 1.81) and type 1 diabetes during the 10-y follow-up (HR: 1.43; 95% CI: 1.04, 1.96) (Table 2), meaning 10-fold increase of relative unit in milk antibody values raises risk of developing islet autoimmunity by 39% and type 1 diabetes by 43%. We also performed the analysis that was restricted to children who had developed outcome before the age of 3 y (HR for multiple islet autoimmunity: 1.64; 95% CI: 1.01, 2.67; HR for type 1 diabetes: 1.67; 95% CI: 1.08, 2.57), and elevated risk of islet autoimmunity and type 1 diabetes associated with the increase in milk antibody concentrations was shown as well.

**TABLE 2:** Risk of islet autoimmunity and type 1 diabetes associated with longitudinal cow milk and casein antibody concentrations from birth to 3 y of age and cord blood cow milk and casein antibody concentrations.

	Multiple islet autoimmunity (*N* = 2069; No. of outcomes = 197)		Type 1 diabetes (*N* = 2069; No. of outcomes = 172)	
Over birth ≤3 y (per 10-fold relative unit change)	HR^1^^,^^2^ (95% CI)	*P*	HR^1^^,^^2^ (95% CI)	*P*
Cow milk IgG	1.39 (1.07, 1.81)	0.014	1.43 (1.04, 1.96)	0.029
Cow milk IgA	1.02 (0.84, 1.25)	0.813	1.03 (0.81, 1.31)	0.798
Casein IgG	1.22 (0.95, 1.57)	0.123	1.01 (0.74, 1.38)	0.960
Casein IgA	1.20 (0.89, 1.60)	0.230	1.14 (0.80, 1.62)	0.474
Cord blood (per 10-fold relative unit change)	HR^2^^,^^3^ (95% CI)	*P*	HR^2^^,^^3^ (95% CI)	*P*
Cow milk IgG	1.30 (1.05, 1.61)	0.014	1.32 (1.02, 1.71)	0.037
Casein IgG	1.47 (1.07, 2.03)	0.019	1.38 (0.93, 2.06)	0.109

1 Adjusted for sex, HLA, region, maternal type 1 diabetes status, and weight *z*-score.

2 Analysis done with Cox time-dependent model. Children followed aged ≤10 y.

3 Adjusted for sex, HLA, region, and maternal type 1 diabetes status.

Further, when we analyzed cord blood separately, cord blood cow milk IgG was associated with higher risk of multiple islet autoimmunity (HR: 1.30; 95% CI: 1.05, 1.61) and type 1 diabetes (HR: 1.32; 95% CI: 1.02, 1.71) as well as cord blood casein IgG with increased risk of multiple islet autoimmunity (1.47; 95% CI: 1.07, 2.03) (Table 2). Treatment group (casein hydrolysate or control formula), duration of breastfeeding, or maternal type 1 diabetes did not modify the associations between cow milk IgG concentrations and risk of multiple islet autoimmunity or type 1 diabetes.

### Consumption of infant formula and dairy products in childhood and risk of multiple islet autoimmunity and type 1 diabetes

Mean amount of infant formula per day was associated with lower risk of multiple islet autoimmunity in all children during the 10-y follow-up (HR: 0.95; 95% CI: 0.91, 1.00) (Table 3). Particularly, amount of formula was protectively associated with multiple islet autoimmunity in the control formula group (HR: 0.92; 95% CI: 0.85, 0.99), whereas not in casein hydrolysate formula group (HR: 0.98; 95% CI: 0.92, 1.05) (Table 3). Maternal type 1 diabetes did not modify the associations. Breastfed infants consumed at 6 mo of age, on average, 1 dL infant formula, whereas in nonbreastfed infants, average intake was >7 dL (Supplemental Table 1). Duration of breastfeeding (categorized as a variable: any breastfeeding <3 mo; 3–5.9 mo; ≥6 mo) was not associated with multiple islet autoimmunity (*P* = 0.105) or type 1 diabetes (*P* = 0.150).

**TABLE 3:** Risk of islet autoimmunity and type 1 diabetes associated with cumulative intake of infant formula and intake frequency of dairy foods over 18 mo to 10 y.

	Multiple islet autoimmunity		Type 1 diabetes	
	HR^1^^,^^2^ (95% CI)	*P*	HR^1^^,^^2^ (95% CI)	*P*
Intake of infant formula per day (per 1 dL intake increase)	*N* = 2069 (No. outcomes = 197)		*N* = 2069 (No. outcomes = 172)	
All children	0.95 (0.91, 1.00)	0.047	0.95 (0.994, 1.000)	0.107
Casein hydrolysate group	0.98 (0.92, 1.05)	0.582	0.98 (0.91, 1.06)	0.618
Control formula group	0.92 (0.85, 0.99)	0.029	0.92 (0.85, 1.01)	0.065
Intake frequency of dairy foods over 18 mo to 10 y (per each time/d)	*N* = 1506 (No. outcomes = 138)		*N* = 1506 (No. outcomes = 118)	
Liquid milk				
All children	1.18 (1.05, 1.33)	0.007	1.06 (0.94, 1.20)	0.363
Casein hydrolysate group^3^	1.28 (1.10, 1.49)	0.017	1.15 (0.98, 1.36)	0.091
Control formula group^3^	1.04 (0.85, 1.26)	0.727	0.95 (0.78, 1.15)	0.598
Children breastfed < 6 mo^4^			0.83 (0.65, 1.06)	0.140
Children breastfed ≥ 6 mo^4^			1.18 (1.02, 1.37)	0.027
Sour milk products (per each time/d)	0.58 (0.42, 0.81)	0.001	0.79 (0.58, 1.06)	0.118
Cheeses (per each time/d)	0.97 (0.75, 1.26)	0.836	1.13 (0.90, 1.42)	0.296

1 Adjusted for sex, HLA, region, and maternal type 1 diabetes status.

2 Children followed aged ≤10 y. Analyses were done with Cox time-dependent model.

3 Treatment group referred to an interaction with intake frequency of liquid milk on risk of islet autoimmunity (*P* = 0.128) and type 1 diabetes (*P* = 0.128).

4 Duration of breastfeeding over 6 mo showed interaction with intake frequency of liquid milk on risk of type 1 diabetes (*P* = 0.014).

Frequency of liquid milk intake from the age 18 mo to 10 y was associated with increased risk of multiple islet autoimmunity (HR: 1.18; 95% CI: 1.05, 1.33), whereas no association was observed for type 1 diabetes (HR: 1.06; 95% CI: 0.94, 1.20) (Table 3). Instead, intake frequency of sour milk products showed association with decreased risk of multiple islet autoimmunity (HR: 0.58; 95% CI: 0.42, 0.81) (Table 3). Furthermore, both treatment group (hydrolysate or control formula) and breastfeeding modified the associations between later intake frequency of liquid milk and risk to develop multiple islet autoimmunity or type 1 diabetes (Table 3). Increased risk was observed particularly in children who had been in casein hydrolysate formula group (HR for islet autoimmunity: 1.28; 95% CI: 1.10, 1.49) or were breastfed for >6 mo (HR for type 1 diabetes: 1.18; 95% CI: 1.02, 1.37) (Table 3).

### Correlations of cow milk antibodies with consumption of infant formula and dairy products

Amount of control formula correlated with all type of cow milk antibodies in both breastfed and nonbreastfed infants, but correlations were higher in breastfed children (Supplemental Table 2). Casein hydrolysate did not show correlations in breastfed infants, whereas in nonbreast infants, casein hydrolysate correlated inversely with all milk antibodies (Supplemental Table 2). Intake frequency of liquid milk at the ages of 18 mo to 3 y correlated with milk antibodies, whereas sour milk products and cheeses mainly did not show correlations (Supplemental Table 3).

## Discussion

The antibody concentrations to cow milk in cord blood and until 3 y of age were found to be linked to higher risk of multiple islet autoimmunity and type 1 diabetes during a 10-y follow-up of genetically high-risk TRIGR children. The consumption of infant formula was associated with reduced risk of multiple islet autoimmunity, especially for those receiving control formula. Conversely, frequent intake of liquid milk after infancy was associated with increased risk of multiple islet autoimmunity or type 1 diabetes, particularly in those children randomly assigned to the casein hydrolysate arm or breastfed for ≥6 mo, i.e., those less exposed to cow milk.

Our findings align with those of the TRIGR pilot study [1] and case-control studies [[2], [3], [4], [5], [6], [7], [8], [9], [10], [11]], suggesting that elevated immune responses to cow milk may indicate an altered intestinal immune system or microbiome, contributing to type 1 diabetes risk. Intestinal inflammation and altered microbiome composition, both bacterial and fungal, has been found in the infants who later develop type 1 diabetes [25,26]. Accordingly, enhanced antibody concentrations to cow milk provide a biomarker for the children with altered immune system prone to develop islet autoimmunity.

In this study, we found that IgG antibodies to cow milk and casein in cord blood were associated with increased risk of multiple islet autoimmunity and type 1 diabetes during follow-up to the age of 10 y at genetically high-risk children. IgG class immunoglobulins in cord blood represent maternal antibodies actively transferred to the fetus by the placenta. Maternal antibodies are also secreted via breastmilk with the purpose to provide immune protection to the offspring [27,28]. Our results suggest that high concentrations of maternal antibodies to cow milk are a marker of risk of type 1 diabetes in the offspring, potentially reflecting the mother’s diet or enhanced immune response to cow milk proteins and, hence, shared genetic characteristics of the intestinal immune system. Further, it could be hypothesized that some harmful mechanism may relate to placentally transferred cow milk IgG antibodies. For example, IgG antibodies can form complexes with the antigen that may trigger priming immune responses already in utero [29]. Furthermore, based on mice models, it has been suggested that placentally transferred IgG antibodies have a strong immunomodulatory potential, particularly during the first 3 wk of life, and they can induce immunologic imprinting with potential long-term effects on the offspring’s immune system [30].

The amount of infant formula was inversely associated with multiple islet autoimmunity risk, particularly in the control formula group. In contrast, frequent postinfancy liquid milk intake was associated with increased risk of multiple islet autoimmunity or type 1 diabetes, particularly for those in the casein hydrolysate formula group or breastfed for >6 mo, i.e., those children who were less exposed to cow milk protein during infancy. Interestingly, sour milk intake showed an inverse association with multiple islet autoimmunity. Cow milk proteins are modified in the processing of sour milk products, e.g., denaturation of proteins occur during the processing. Our study suggests that the formulation and timing of cow milk exposure may impact risk differently, being either immunogenic or tolerogenic. Thus, it is interesting to speculate that cow milk could contain an antigen that specifically triggers islet autoimmunity. If this is the case, our results of this study suggest that it may be beneficial to be exposed to cow milk protein by 6 mo of age for the development of immunologic tolerance. To our knowledge, these are the first findings to suggest that the consumed amount of cow milk–based infant formula is linked to lower risk of islet autoimmunity. In the Finnish Type 1 Diabetes Prediction and Prevention (DIPP) study cohort, the amount of infant formula was not associated with risk of islet autoimmunity [18]. To our knowledge, other prospective studies have investigated the association of the exposure age to cow milk–based infant formula, but not the amount of formula, and risk of developing islet autoimmunity or type 1 diabetes with inconsistent results [14,15,17,19,[31], [32], [33], [34], [35], [36], [37], [38], [39]]. Our observation that frequent postinfancy intake of liquid milk was associated with increased risk of islet autoimmunity is in accordance with results of several previous prospective studies [[13], [14], [15], [16], [17], [18],^33^].

In our study, as expected, control cow milk–based infant formula intake correlated directly with milk antibody concentrations, whereas intake of casein hydrolysate formula showed inverse correlation in nonbreastfed infants and no associations was observed in breastfed infants. Although infancy liquid milk intake correlated with milk antibody concentrations, sour milk and cheeses showed no correlations. Altogether, our results suggest that liquid milk consumption in later childhood, but not in infancy, may partly explain the present associations observed between enhanced immune responses to cow milk and increased risk of multiple islet autoimmunity.

The strengths of this study include a unique sample set from a large, international double-blind randomized infant feeding trial with standardized, prospective, and frequent collection of dietary data and good dietary compliance [40,41]. We had a possibility to study both multiple islet autoimmunity and clinical disease as outcomes. One strength was that the milk antibody measurements were comprehensively available up to age 3 y and we had the possibility to analyze 4 different cow milk antibody types. However, a limitation was that antibodies to many individual milk proteins (bovine serum albumin, β-lactoglobuline, or bovine insulin), which have been related to type 1 diabetes in previous studies, were not available. Additionally, we did not measure antibodies to food proteins other than milk proteins, a limitation given that all individuals produce IgG antibodies against food proteins. However, previous case-control studies have examined antibodies to egg protein ovalbumin [24] and wheat and rye protein gliadin [42], revealing similar concentrations in both cases and controls, and only milk antibody concentrations were found to be higher in children with type 1 diabetes than those in controls. These results suggests that increased immunologic reactivity specifically to cow milk may be associated with type 1 diabetes. One additional limitation in our study was that the food frequency questionnaire used to assess milk consumption from 18 mo to 10 y was not validated, and consumption of dairy products could be analyzed only by intake frequencies (not total amounts). Further, milk intake analysis could not be adjusted for energy intake because total diet was not assessed. However, in our data, intake frequency of liquid milk correlated with the milk antibody concentrations, suggesting that the food frequency questionnaire was a valid method to estimate milk consumption. Generalizability of our results to the whole population remains open because our study population comprised high-risk children owing to their HLA-conferred genetic risk of type 1 diabetes and having a first-degree relative/s with type 1 diabetes.

In conclusion, our results support the view that both increased humoral immune responses to cow milk proteins and frequent intake of liquid milk after infancy are associated with higher risk of developing type 1 diabetes in children genetically predisposed to develop type 1 diabetes. Enhanced antibody concentrations to cow milk may provide a biomarker of immune system prone to develop islet autoimmunity.

## Author contributions

The authors’ responsibilities as follows – SN, SMV: conceived and designed the study and final content of the manuscript; ES, MK, JK, SMV, AN, OV: were responsible for the acquisition of data; ES, MK: performed the laboratory analysis; DC: carried out the statistical analyses; SN, SMV, MM, LH: drafted the manuscript with contributions from ES, HH, OV, MK, and TEK; MK, SMV: are the guarantors of this work; SMV: is the guarantor of this work and, as such, had full access to all the data in the study and takes responsibility for the integrity of the data and the accuracy of the data analysis; and all authors: contributed to the interpretation of the data and have read and approved the manuscript and critically reviewed and approved the version to be published.

## Conflict of interest

The authors report no conflicts of interest.

## Funding

This work was supported by National Institutes of Health (grants 1DP3DK106918-01, HD040364, HD042444, and HD051997), the Eunice Kennedy Shriver National Institute of Child Health and Development (NICHD), National Institute of Diabetes and Digestive and Kidney Diseases, Canadian Institutes of Health Research, JDRF, the Commission of the European Communities (specific RTD programme Quality of Life and Management of Living Resources, contract QLK1-2002-00372 Diabetes Prevention), the European Foundation for the Study of Diabetes/JDRF/Novo Nordisk Focused Research Grant, Academy of Finland (Centre of Excellence in Molecular Systems Immunology and Physiology Research 2012-2017, Decision No. 250114, Decision No. 339922), Dutch Diabetes Research Foundation, and Finnish Diabetes Research Foundation. The study sponsors were not involved in the design of the study; the collection, analysis, and interpretation of data; writing the report; or the decision to submit the report for publication.

## Data availability

No online repository for TRIGR data is currently available. Information is available on reasonable request from the TRIGR Central Coordination Committee led by Dr Mikael Knip from the University of Helsinki, Finland. The authors confirm that, for approved reasons, some access restrictions apply to the datasets generated during and/or analyzed during the current study underlying the findings. The researchers interested in using the data are required to follow the terms of a number of clauses designed to ensure the protection of privacy and compliance with relevant regulation. Data are available on request due to ethical restrictions, pending approval from the relevant ethical committees.
