# Supplementary material for: High Concentrations of Immunoglobulin G Against Cow Milk Proteins and Frequency of Cow Milk Consumption Are Associated With the Development of Islet Autoimmunity and Type 1 Diabetes—The Trial to Reduce Insulin-dependent Diabetes Mellitus (IDDM) in the Genetically at Risk (TRIGR) Study
Source: J Nutr. 2024 Jun 19;154(8):2493–500. doi: 10.1016/j.tjnut.2024.06.005 (PMC11375457; doi:10.1016/j.tjnut.2024.06.005)
Supplement: Multimedia component 1 [file mmc1.docx]

**Supplementary**

**Supplementary Figure 1.** The participant flow chart.

**Supplementary Table 1**. Amount of infant formula consumption (mean and SD) at the age of 3 and 6 month by breastfeeding.

|  | Daily consumption of study formula (liters)  Mean (SD) |
| --- | --- |
| **At 3 months** |  |
| ***Breastfed*** |  |
| Casein hydrolysate n=660 | 0.05 (0.16) |
| Control formula n=643 | 0.08 (0.21) |
| ***Non-breastfed*** |  |
| Casein hydrolysate n=258 | 0.71 (0.42) |
| Control formula n=272 | 0.72 (0.37) |
| **At 6 months** |  |
| ***Breastfed*** |  |
| Casein hydrolysate n=545 | 0.09 (0.20) |
| Control formula n=492 | 0.10 (0.22) |
| ***Non-breastfed*** |  |
| Casein hydrolysate n=352 | 0.71 (0.45) |
| Control formula n=393 | 0.75 (0.43) |

**Supplementary Table 2**. Spearman correlations between amount of casein hydrolysate or control formula consumed and antibodies to cow’s milk at respective age among breastfed and non-breastfed infants.

| Amount of study formula consumption at visit | Correlation with cow’s milk IgG | p-value | Correlation with  cow’s milk IgA | p-value | Correlation with  casein IgG | p-value | Correlation with  casein IgA | p-value |
| --- | --- | --- | --- | --- | --- | --- | --- | --- |
| **At 3 mo** |  |  |  |  |  |  |  |  |
| ***Breastfed*** *(n=1283)* |  |  |  |  |  |  |  |  |
| Casein hydrolysate (n=640) | 0.021 | 0.589 | 0.017 | 0.666 | 0.003 | 0.939 | 0.016 | 0.690 |
| Control formula (n=643) | **0.555** | **<0.001** | **0.668** | **<0.001** | **0.540** | **<0.001** | **0.595** | **<0.001** |
| ***Non-breastfed*** *(n=530)* |  |  |  |  |  |  |  |  |
| Casein hydrolysate (n=258) | **-0.175** | **0.005** | **-0.169** | **0.006** | **-0.218** | **<0.001** | **-0.124** | **0.047** |
| Control formula (n=272) | **0.252** | **<0.001** | **0.267** | **<0.001** | **0.262** | **<0.001** | **0.278** | **<0.001** |
| **At 6 mo** |  |  |  |  |  |  |  |  |
| ***Breastfed*** *(n=1036)* |  |  |  |  |  |  |  |  |
| Casein hydrolysate (n=545) | **0.098** | **0.022** | 0.080 | 0.062 | 0.052 | 0.228 | **0.095** | **0.026** |
| Control formula (n=491) | **0.559** | **<0.001** | **0.428** | **<0.001** | **0.468** | **<0.001** | **0.335** | **<0.001** |
| ***Non-breastfed*** *(n=745)* |  |  |  |  |  |  |  |  |
| Casein hydrolysate (n=352) | **-0.236** | **<0.001** | **-0.174** | **0.001** | **-0.192** | **<0.001** | **-0.129** | **0.016** |
| Control formula (n=393) | **0.197** | **<0.001** | **0.204** | **<0.001** | **0.170** | **<0.001** | **0.194** | **<0.001** |

**Supplementary Table 3**. Spearman correlations between frequencies of cow’s milk product intake at 18 months up to 3 years of age and antibodies to cow’s milk at respective age.

| Intake frequency (times per day) | Correlation with cow’s milk IgG | p-value | Correlation with cow’s milk IgA | p-value | Correlation with Casein IgG | p-value | Correlation with Casein IgA | p-value |
| --- | --- | --- | --- | --- | --- | --- | --- | --- |
| Liquid milk |  |  |  |  |  |  |  |  |
| 18 mo (n=583) | **0.292** | **<0.001** | **0.309** | **<0.001** | **0.228** | **<0.001** | **0.288** | **<0.001** |
| 2 y (n=554) | **0.329** | **<0.001** | **0.349** | **<0.001** | **0.244** | **<0.001** | **0.304** | **<0.001** |
| 3 y (n=357) | **0.265** | **<0.001** | **0.262** | **<0.001** | **0.202** | **<0.001** | **0.225** | **<0.001** |
| Sour milk products |  |  |  |  |  |  |  |  |
| 18 mo (n=584) | -0.036 | 0.390 | -0.033 | 0.429 | -0.042 | 0.308 | -0.073 | 0.078 |
| 2 y (n=555) | 0.021 | 0.615 | 0.032 | 0.446 | -0.013 | 0.766 | -0.006 | 0.897 |
| 3 y (n=357) | -0.008 | 0.876 | -0.012 | 0.817 | -0.017 | 0.746 | -0.012 | 0.821 |
| Cheeses |  |  |  |  |  |  |  |  |
| 18 mo (n=584) | 0.060 | 0.150 | 0.050 | 0.230 | 0.048 | 0.244 | 0.058 | 0.159 |
| 2 y (n=553) | 0.066 | 0.119 | 0.036 | 0.396 | **0.105** | **0.013** | 0.020 | 0.639 |
| 3 y (n=357) | 0.069 | 0.192 | -0.033 | 0.530 | 0.041 | 0.440 | -0.051 | 0.341 |
